# Hospital Conditions in London Sixty Years Ago

**Published:** 1921-09-24

**Authors:** 


### A BUNDLE OF OLD REPORTS.

### Hospital Conditions in London Sixty Years Ago.

An enthusiastic administrator of a hospital which was opened in London about sixty years ago—now one of the most important of its kind in the world—collected together the reports of many of the chief hospitals of the Metropolis of that period, no doubt with a view to seeking guidance from the matured experience of other institutions. These reports are all round about the years 1859 to 1861, long before the Uniform System put an end to an endless variety of presenting hospital facts and figures to the public.

A few weeks ago the readers of The Hospital were informed of the retirement of Mr. Quennell, who had been the Secretary of Westminster Hospital from almost before the memory of living administrators, but the report before us is so ancient that it contains the name of his predecessor, Mr. F. J. Wilson. We also note amongst the list of officers the name of Dr. Charles B. Radcliffe, who was also one of the earliest physicians of the National Hospital for the Paralysed, Queen Square. Another officer is described as "cupper and collector"; this gentleman no doubt was called upon to bleed people by two distinct methods. The district served by the Westminster is described as composed of densely populated parishes, and, as St. Thomas's had not then taken up its position opposite the House of Commons, the poor of Lambeth sought relief in emergency across the river.

The income and expenditure account of the hospital is arranged in a curious way, but to some extent approximates to present-day methods. The expenditure was divided into four main headings—namely, house expenses (*i.e.* food, hardware, coals, etc.), dispensary and surgery, miscellaneous expenses, and repairs. One curious item is the sum of £6 8s. 6d. paid to wine coopers for removing and binning 1,000 bottles of sherry wine presented by his Grace the Duke of Northumberland. The expenditure on thermometers was only 6s. In the year in question there were 1,539 in-patients and 18,180 out-patients treated at a cost of £5,663. The number of patients dealt with in a recent year was 1,834 in-patients and 13,903 out-patients, and the cost £34,993.

### Profuse Use of Stimulants.

There are two common features in many of the reports in this collection. One is that the resident medical staff all paid something for their board and lodging, and another that the expenditure on wines and malt liquors was much heavier than at the present time. At the Westminster, for instance, the beer bill was £208 (the cost of milk only £120), while under the dispensary account there is an expenditure of £267 for wine, gin, and brandy.

At the London Hospital several names borne by the patrons and committee-men still appear in present-day reports, such as de Rothschild, Hoare, Buxton, Gurney, Sebag, Charrington, etc. We notice amongst the assistant surgeons Mr. George Crichett, who, if we mistake not, was father of Sir George Anderson Crichett, the well-known ophthalmic surgeon. Mr. William John Nixon had been Secretary since 1846. This gentleman was afterwards pensioned and lived to a great age, and his son was Secretary of University College Hospital. During 1861, of 3,788 cases, 323 died. In the table detailing the percentages of deaths there are some curious explanatory remarks referring to bygone years. For instance, 1837 is described as the year "when the new sewers were completed," and 1840 as the era of hot air being discontinued in the wards; 1849 and 1854, when the deaths were not above the average, were cholera years. The accounts of the hospital are not given very fully. House expenses, provisions, etc., not detailed in any way, amounted to £7,177. Out of a total expenditure of £18,942, £673 was spent on wine, brandy, and gin, and £1 10s. on taxes The House Governor's apartments were done up in this year, and accounted for part of an outlay on repairs of £2,044.

The part of the report devoted to the Samaritan Society is preceded by a circular engraving by F. Bartolozzi, R.A., after a design by E. Edwards depicting the parable of the Good Samaritan.

From the frontispiece of the annual report of St. George's Hospital for 1861, the building was apparently practically the same in those days as it is now, subsequent alterations having been made to that part which is not visible from the street. We notice amongst the list of medical officers the name of Mr. Timothy Holmes as assistant surgeon. Many hospital workers will remember this gentleman, who later became Treasurer of the hospital. The Surgical Registrar was Mr. Pickering Pick, the Secretary Mr. W. J. Taylor, and Mr. Todd, the Steward and Clerk, is no doubt the gentleman who subsequently held the office of Secretary for a very long time. The annual report mentions a large legacy from Mr. Morley, payable in 1863, to be devoted to the building of a convalescent branch. There was apparently a deficit in 1861 of over £4,000, which was made up by the sale of stock, Consols selling at the satisfactory price of 92¼. Amongst the payments it is noticed that extra diets are separated from other things, and are detailed as stout, jelly, biscuits, etc. £98 was spent on beer and £437 on wines and spirits. There were 3,436 in-patients and 10,736 out-patients during the year.

### Controversy on a Hospital's Removal.

In 1862 the authorities of St. Thomas's Hospital were considering the removal or rebuilding of the hospital. It was generally felt that removal was advisable, but the position of the new site was a matter of great debate, and correspondence on the subject was carried on in *The Times* newspaper. The medical officers made strong representations to the Governors that the intentions of the founder of the charity would be defeated and the priceless boon of a good school sacrificed should physicians and surgeons of high Metropolitan standing find it difficult to attend. On the other hand, Mr. R. G. Whitfield, who was R.M.O., wrote at great length advocating removal to Lewisham. As a sample of his literary style the following may be quoted: "The *dilletanti parvenus* of the present day, or quasi-fellows of learned societies, may presume in their own love of self to suppose that an humble apothecary cannot be actuated otherwise than by the same self-interest. I am not ashamed to acknowledge that I am 'the solitary medical advocate' for the removal of St. Thomas's to a more distant site if its present destruction is inevitable."

At this time the Government was thinking of removing their offices from Somerset House, and it was supposed that the authorities were not against this building being converted into a hospital (*i.e.* St. Thomas's) in conjunction with King's College. The proposition met with the approval of the medical staff, but how it was finally disposed of does not transpire. Another possibility was an amalgamation with Bethlem, then as now in proximity to the present site of St. Thomas's. Mr. Whitfield, in his clever piece of special pleading on behalf of Lewisham, pointed out that the railway station was but five miles from London Bridge, and only about half a mile from the site he suggested, the directors of the North Kent Line having agreed to allow a siding for the express use of the hospital. The gravel soil and the giddy eminence of 86 feet above mean water-mark are mentioned as excellent reasons for choice, and a table of mortality was presented showing that Lewisham was in those days apparently the healthiest part of London, the death-rate being only 17 in the 1,000. This table is such a convincing piece of evidence that one has strong suspicions that it does not contain exhaustive information. Thirty-five districts are mentioned : Lewisham is shown as the healthiest, and St. Saviour's, Southwark, the parish in which St. Thomas's Hospital was then, the unhealthiest.

Old King's College Hospital.

The report of King's College, Portugal Street, for 1860 gives the name of the Secretary of the day as Mr. James S. Blyth. There was a Mr. J. F. Blyth who was Secretary of the Royal Free Hospital in the 'eighties, and possibly he was a connection. Mr. William H. Smith, jun., was a member of the committee of management, this gentleman no doubt being the head of the great news-distributing business, and the father of the present Chairman of King's College. We see that Mr. Robert Williams gave £1,000 to the hospital in 1850. He was a member of Messrs. Williams Deacon and Co., who have been connected with voluntary hospitals for a great many years. The accounts of King's College were noticeably nearer the Uniform System than those of any of the other hospitals of the time that we have examined. The expenditure headings are : Provisions, domestic expenses (washing, heating, lighting, bedding, etc.), surgery and dispensary, salaries, repairs and miscellaneous. £316 was spent on porter and ale, and £6 16s. on herbs and leeches; £250 was paid to the hospital for board of the medical officers. The accounts of the building fund show that cash had been received to date of approximately £100,000 for the new building, which was commenced shortly afterwards. The total expenses on the current account were £8,384, and 1,622 in-patients and 28,000 out-patients were treated.

We notice on the list of officers both at the Middlesex and University College Hospitals there was a "cupper" in the person of Mr. H. C. Betts. At the Middlesex Dr. Henry Thompson was one of the physicians, and at University College Hospital Mr. R. Quain, F.R.S., was senior surgeon. Mr. Alexander Shedden was Secretary of the former hospital, and Mr. J. W. Goodiff, Clerk to the Committee of the latter. At both hospitals the cost for beer and wine and spirits was very high.

It is very seldom nowadays that one hospital makes capital in its appeal out of the prosperity of another hospital. In this respect things possibly have changed, but the report of University College Hospital alludes to the richly endowed hospitals of Guy's and St. Thomas's having ample funds to meet heavy calls arising from the vicinity of the railways south of the Thames, while University College Hospital depends entirely on the help given by the public. The Benevolent Society at University College Hospital was under the matron, Miss Robottom, and apparently discharged the functions of the ordinary Samaritan Society, sending patients to the seaside and providing clothes for destitute patients.

These are but cursory notes upon a mass of material which might very well deserve more extended notice, but may lead others to study old records and bring to light features in the development of the voluntary system.

